# Transcriptomic profiling of murine macrophages during *Francisella tularensis* infection

**DOI:** 10.1128/mra.00856-26

**Published:** 2026-09-21

**Authors:** Ma'ayan Israeli, Inbar Cohen-Gihon, Ofir Israeli, Ofer Cohen, Adi Bercovich-Kinori

**Affiliations:** 1 Department of Biochemistry and Molecular Genetics, Israel Institute for Biological Research 54623, Ness-Ziona, Israel; Wellesley College, Wellesley, Massachusetts, USA

**Keywords:** RNA-seq, tularemia

## Abstract

*Francisella tularensis*, a virulent bioterrorism threat, subverts host immune responses within macrophages, its primary replication niche. To elucidate the temporal dynamics of the host response, we announce comprehensive time-course RNA-seq data sets capturing the global transcriptional profile of murine RAW 264.7 macrophages across early and late stages of *F. tularensis* infection.

## ANNOUNCEMENT

*Francisella tularensis* is a highly pathogenic gram-negative bacterium distinguished by its ability to cause lethal disease with an infectious dose of as few as 10 to 15 organisms (1). This extreme infectivity, combined with its capacity for aerosol dissemination, has led to its classification as a Tier 1 Category A select agent and a significant bioterrorism concern (2–6). Its pathogenesis relies on evading and actively suppressing host immune responses (7–9). However, a high-resolution, time-dependent understanding of this host manipulation remains incomplete. To address this gap, we present a comprehensive RNA-seq data set mapping the transcriptional profile of murine RAW 264.7 macrophages during both early and late stages of *F. tularensis* infection.

GFP-expressing *Francisella tularensis* subsp. *holarctica* LVS (American Type Culture Collection [ATCC] 29684), an attenuated strain excluded from the Select Agent list, was used to infect RAW264.7 cells (ATCC TIB-71).

Bacterial cultures were grown for 16 h at 37°C to mid-log phase in TSBC (TSB Difco, supplemented with 0.1% cysteine, BD, France). The bacteria were washed with phosphate-buffered saline (PBS) and re-suspended in Dulbecco's Modified Eagle Medium (DMEM) supplemented with 10% heat-inactivated fetal bovine serum. To capture both immediate and delayed transcriptional dynamics, cells were infected in biological triplicates, with the live vaccine strain (LVS) culture at an MOI of 2,500, and incubated for 0.5, 1.5, 4, 8, 24, and 48 h at 37°C. For later time points (>3 h post-infection), cells were treated with gentamicin to eliminate extracellular bacteria. Following incubation, mock (designated as wt *t* = 0) and infected samples were washed with PBS and subjected to RNA extraction using the RNeasy kit (Qiagen) according to the manufacturer’s instructions, while residual DNA was digested using RNase-free DNase (Qiagen). Of the 21 RNA samples assessed, 20 passed quality control (one 48-h replicate was excluded due to insufficient RNA quality).

RNA-seq was performed by CD Genomics (Shirley, NY, USA). Sequencing libraries were generated using standard Illumina Stranded mRNA library preparation protocols according to the manufacturer’s instructions. Paired-end sequencing (2 × 100 bp) was performed on the Illumina NovaSeq 6000 system. Raw reads were preprocessed with fastp v1.0.1 (-q 25, -l 36), which performed default paired-end adapter trimming and removed reads shorter than 36 nt or containing >40% bases below Phred 25 (10, 11). Data quality was subsequently assessed using FastQC v0.12.1 (12). Clean reads were aligned to the mouse reference genome (GRCm39) using HISAT2 v2.2.1 (13). Gene expression levels were quantified as read counts using featureCounts v2.1.1 (14). Analysis of differentially expressed genes under various conditions was performed using the R package DESeq2 v1.46.0, with default parameters (15). Sequencing yielded 44.8 to 77.7 M reads per sample (Table 1), with mapping rates ranging between 87.8 and 94.9% across all samples.

**TABLE 1:** Summary of transcriptome samples submitted

Sample^*a*^	No. of reads	% mapped reads^*b*^	GEO accession no.^*c*^	SRA accession no.
wt_t0 rep1	47,200,584	93.2	GSM9863375	SRX34209077
wt_t0 rep2	45,299,670	92.7	GSM9863376	SRX34209078
wt_t0 rep3	46,820,220	93.7	GSM9863377	SRX34209079
wt_t0.5 rep1	47,660,822	93.7	GSM9863378	SRX34209080
wt_t0.5 rep2	47,937,478	94.4	GSM9863379	SRX34209081
wt_t0.5 rep3	44,770,780	93.8	GSM9863380	SRX34209082
wt_t1.5 rep1	45,663,122	94.5	GSM9863381	SRX34209083
wt_t1.5 rep2	46,139,848	94.6	GSM9863382	SRX34209084
wt_t1.5 rep3	46,737,672	95.2	GSM9863383	SRX34209085
wt_t4 rep1	45,674,644	94.7	GSM9863384	SRX34209086
wt_t4 rep2	45,642,700	96.0	GSM9863385	SRX34209087
wt_t4 rep3	46,484,070	94.7	GSM9863386	SRX34209088
wt_t8 rep1	46,225,892	94.9	GSM9863387	SRX34209089
wt_t8 rep2	45,778,010	94.9	GSM9863388	SRX34209090
wt_t8 rep3	47,151,552	94.8	GSM9863389	SRX34209091
wt_t24 rep1	46,657,418	94.5	GSM9863390	SRX34209092
wt_t24 rep2	45,667,836	94.1	GSM9863391	SRX34209093
wt_t24 rep3	45,835,310	94.1	GSM9863392	SRX34209094
wt_t48 rep1	76,806,488	88.2	GSM9863393	SRX34209095
wt_t48 rep2	77,758,304	87.8	GSM9863394	SRX34209096

^*a*^ Biological replicated samples are denoted as rep1, rep2, and rep3.

^*b*^ % of reads mapped to reference genome: mouse: GRCm39.

^*c*^ The GEO accession number of the transcriptome series is GSE337952. SRA Bioproject: PRJNA1491986. The GEO title of the project is “Transcriptome Profiling of Murine Macrophages During *Francisella tularensis *Infection”.

By capturing both immediate and delayed transcriptional changes, this study offers a time-course transcriptome resource illuminating how specific host factors drive the longitudinal pathophysiology of tularemia.

## Data Availability

The transcriptomic data have been deposited to the NCBI database, and their GEO accession numbers are provided in Table 1.
